# Corrigenda for three related articles

**DOI:** 10.1107/S2056989015003564

**Published:** 2015-03-07

**Authors:** P. L. Nilantha Lakshman

**Affiliations:** a Department of Food Science and Technology, University of Ruhuna, Mapalana, Kamburupitiya 81100, Sri Lanka

## Abstract

The schemes and chemical names are corrected in three related papers: Vishnupriya, Suresh, Bharkavi *et al.* [*Acta Cryst.* (2014), E**70**, o968–o969], Vishnupriya, Suresh, Gunasekaran *et al.* [*Acta Cryst.* (2014), E**70**, o978], and Vishnupriya, Suresh, Sakthi *et al.* [*Acta Cryst.* (2014), E**70**, o1120–o1121].

## Abstract 2

Corrigenda for three articles.

In the paper by Vishnupriya, Suresh, Bharkavi *et al.* (2014 ▸), the chemical name in the title should be given as ‘4-(2-bromo­phen­yl)-2-(1*H*-indol-3-yl)-6-(thiophen-2-yl)­pyridine-3-carbo­nitrile’ and the correct scheme is shown below.
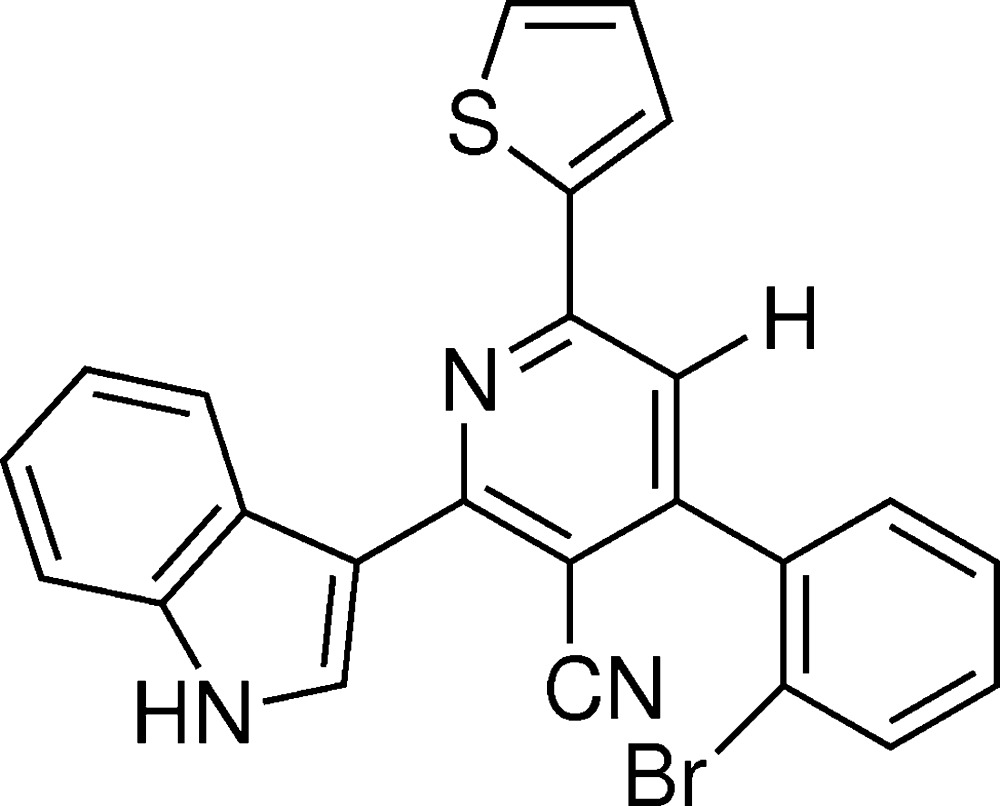


In the paper by Vishnupriya, Suresh, Gunasekaran *et al.* (2014 ▸), the chemical name in the title should be given as ‘4-(4-chloro­phen­yl)-2-(1*H*-indol-3-yl)-6-phenyl­pyridine-3-carbo­nitrile’ and the correct scheme is shown below.
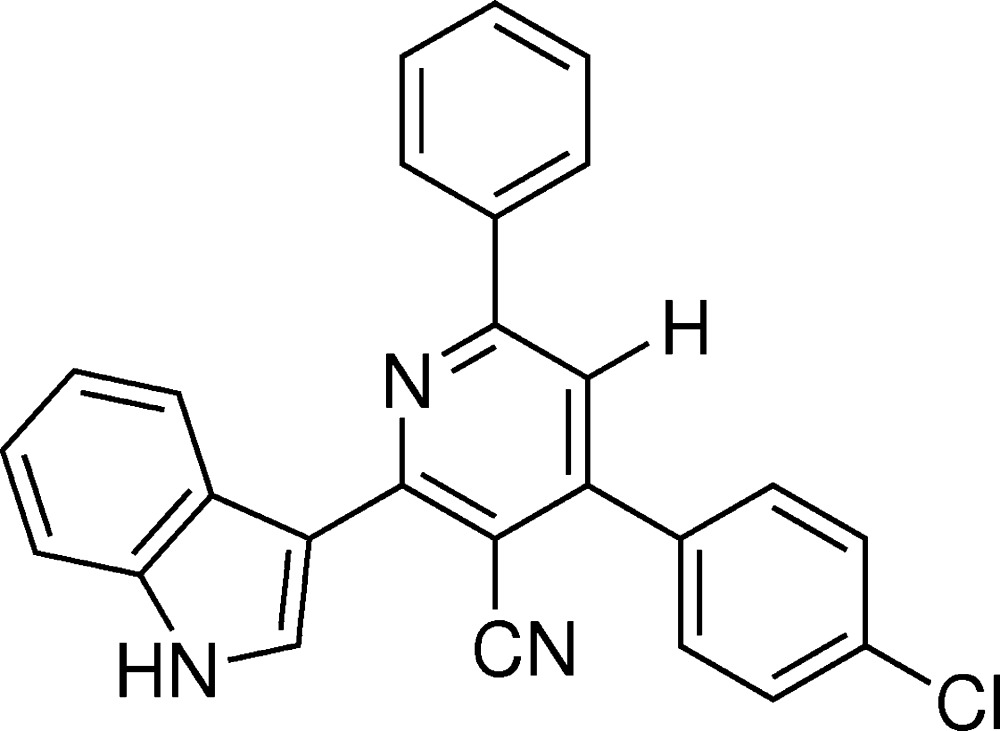


In the paper by Vishnupriya, Suresh, Sakthi *et al.* (2014 ▸), the chemical name in the title should be given as ‘2-(1*H*-indol-3-yl)-4-(4-meth­oxy­phen­yl)-6-phenyl­pyridine-3-carbo­nitrile’ and the correct scheme is shown below.
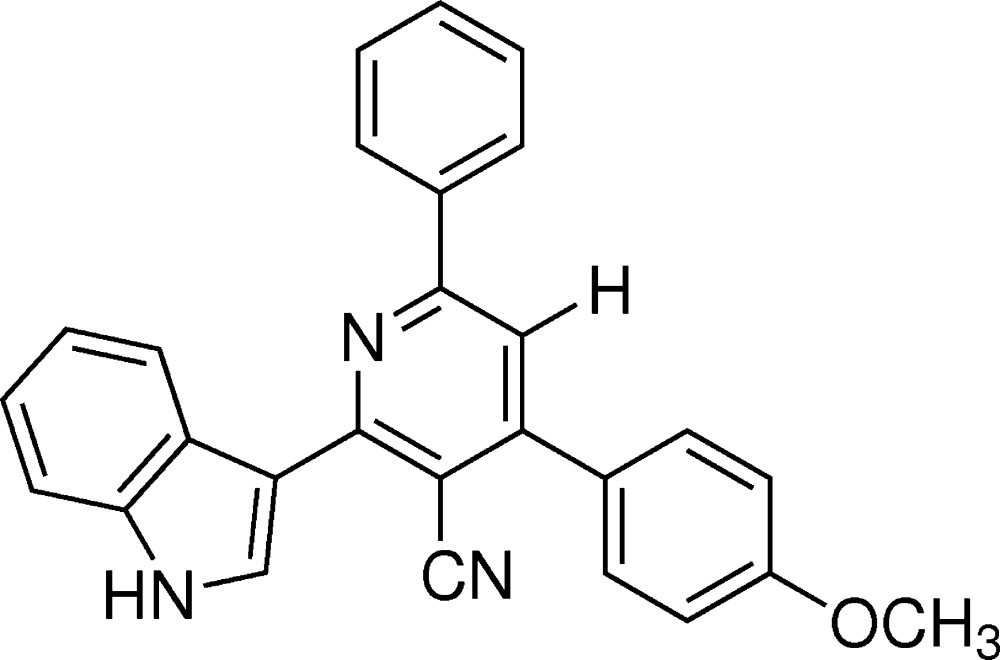


## References

[bb1] Vishnupriya, R., Suresh, J., Bharkavi, S., Perumal, S. & Lakshman, P. L. N. (2014). *Acta Cryst.* E**70**, o968–o969. 10.1107/S1600536814017188 PMC4186133 25309284

[bb2] Vishnupriya, R., Suresh, J., Gunasekaran, P., Perumal, S. & Lakshman, P. L. N. (2014). *Acta Cryst.* E**70**, o978. 10.1107/S1600536814017693 PMC4186124 25309290

[bb3] Vishnupriya, R., Suresh, J., Sakthi, M., Perumal, S. & Lakshman, P. L. N. (2014). *Acta Cryst.* E**70**, o1120–o1121. 10.1107/S1600536814020170 PMC4257225 25484706

